# A potential role for the cerebellar nuclei in absence seizures

**DOI:** 10.1186/1471-2202-14-S1-P170

**Published:** 2013-07-08

**Authors:** Parimala Alva, Lieke Kros, Reinoud Maex, Chris I De Zeeuw, Rod Adams, Neil Davey, Volker Steuber, Freek E Hoebeek

**Affiliations:** 1 Science and Technology Research Institute, University of Hertfordshire, Hatfield, AL10 9AB, UK; 2 Department of Neuroscience, Erasmus Medical Center, Rotterdam, The Netherlands; 3 Department of Cognitive Sciences, École Normale Supérieure, Paris 75005, France

## 

Absence seizures are characterized by a temporary lapse of consciousness, which typically lasts up to ten seconds, and they are accompanied by spike-wave discharges (SWDs) in cerebral electroencephalogram (EEG) recordings. The oscillatory activity that underlies cortical SWDs has been shown to often originate from a specific focus that can be located in various brain regions, such as the cerebral cortex, thalamus or hippocampus [1]. Yet, the role of the cerebellum, which is anatomically connected to each of these potential foci, is unknown. Here, we used *Cacna1a^tottering ^(tg) *mice, an established model for absence epilepsy characterized by a loss-of-function of calcium channels [3], to study how the cerebellar activity changes during absence seizures. Given that recent evidence shows that cerebellar Purkinje cells in *tottering *mice exhibit an altered expression of calcium channels [2] and structurally abnormal synapses in the cerebellar nuclei (CN), it is our hypothesis that the cerebellar output, which is dominated by neurons in the CN, changes during absence seizures.

**Figure 1:**
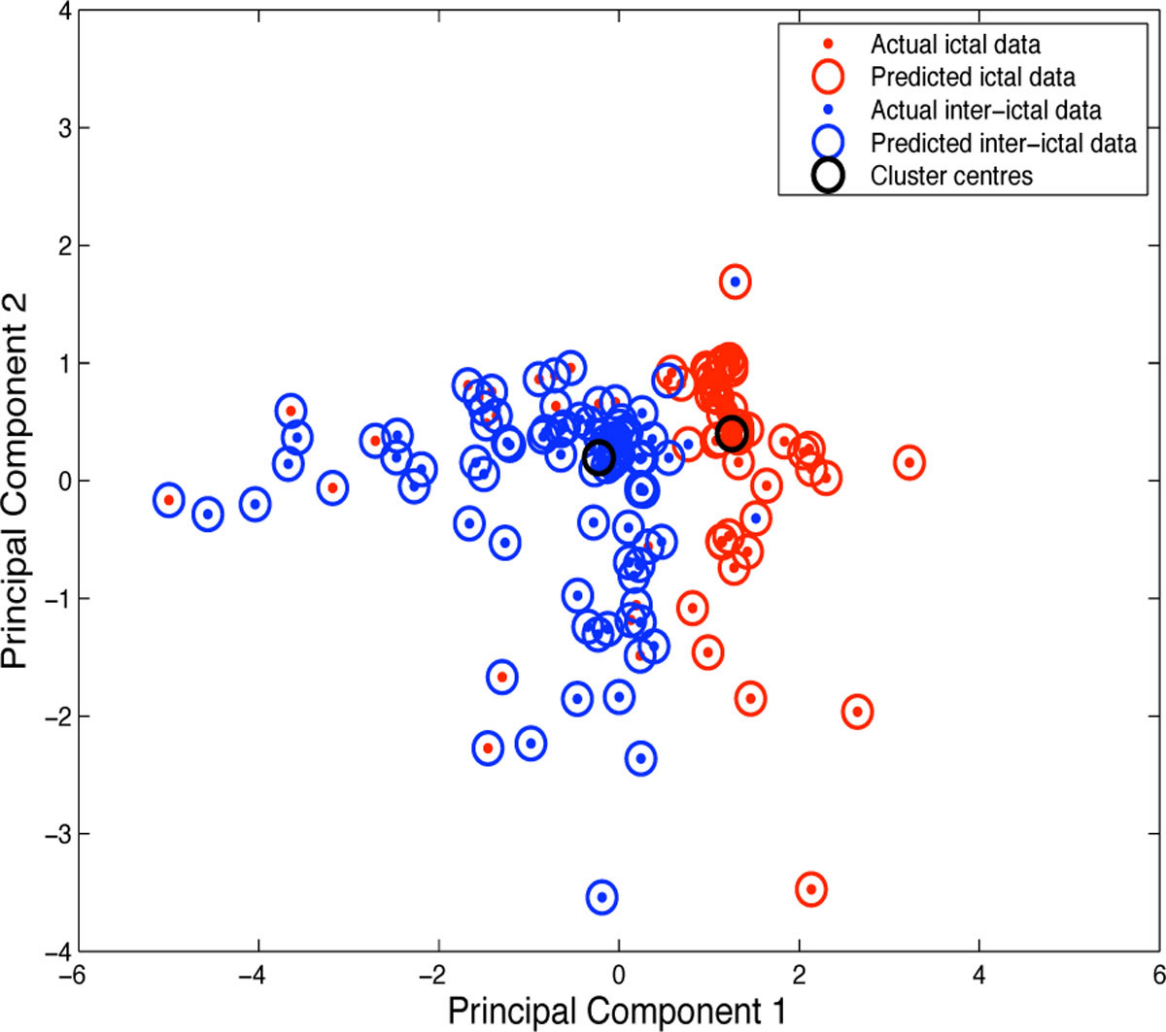
**K-Means clustering showing two distinct clusters for ictal and inter-ictal data**. The two variables, principal component 1 and principal component 2 are derived by PCA of CV, CV2 and PE of ictal and inter-ictal data.

In the present study, we analysed extracellular spike trains of CN units, and simultaneous EEG recordings, in ten awake head-restrained mice. The recordings were partitioned into equal-length segments of 900 ms and, depending on whether spike-wave discharges occurred in the EEG or not, considered as ictal or inter-ictal data. The metrics considered for analysis were the CV, CV2, firing rate and permutation entropy (PE). When a one-dimensional analysis of the metrics for ictal and inter-ictal data was conducted, it was noted that a single metric was not sufficient to differentiate between the ictal and inter-ictal data. Therefore, the three variables, CV, CV2 and PE, were combined to form two new variables, Principal Component 1 and Principal Component 2 (Figure 1), using Principal Component Analysis (PCA). The two new variables, Principal Component 1 and Principal Component 2, were then subjected to cluster analysis using K-Means clustering having two centers. Firing rate was not considered for PCA and clustering as better results were achieved while excluding it.

The result, shown in Figure 1, depicts two distinct clusters for ictal and inter-ictal data. The confusion matrix for this data denotes true positive where predicted ictal data match actual ictal data (95.7%), true negative where predicted inter-ictal data match actual inter-ictal data (70%), false positive for actual ictal data incorrectly predicted as inter-ictal data (4.3%), and false negatives for actual inter-ictal data incorrectly predicted as ictal data (30%). The F-score achieved by this classification was 0.84. The separation between the ictal and inter-ictal data could further be improved by the application of Support Vector Machines (SVMs). We are currently using a conductance based model of a CN neuron to study which conditions can result in spike patterns associated with seizures.
